# Stopping randomized trials early for benefit: a protocol of the Study Of Trial Policy Of Interim Truncation-2 (STOPIT-2)

**DOI:** 10.1186/1745-6215-10-49

**Published:** 2009-07-06

**Authors:** Matthias Briel, Melanie Lane, Victor M Montori, Dirk Bassler, Paul Glasziou, German Malaga, Elie A Akl, Ignacio Ferreira-Gonzalez, Pablo Alonso-Coello, Gerard Urrutia, Regina Kunz, Carolina Ruiz Culebro, Suzana Alves da Silva, David N Flynn, Mohamed B Elamin, Brigitte Strahm, M Hassan Murad, Benjamin Djulbegovic, Neill KJ Adhikari, Edward J Mills, Femida Gwadry-Sridhar, Haresh Kirpalani, Heloisa P Soares, Nisrin O Abu Elnour, John J You, Paul J Karanicolas, Heiner C Bucher, Julianna F Lampropulos, Alain J Nordmann, Karen EA Burns, Sohail M Mulla, Heike Raatz, Amit Sood, Jagdeep Kaur, Clare R Bankhead, Rebecca J Mullan, Kara A Nerenberg, Per Olav Vandvik, Fernando Coto-Yglesias, Holger Schünemann, Fabio Tuche, Pedro Paulo M Chrispim, Deborah J Cook, Kristina Lutz, Christine M Ribic, Noah Vale, Patricia J Erwin, Rafael Perera, Qi Zhou, Diane Heels-Ansdell, Tim Ramsay, Stephen D Walter, Gordon H Guyatt

**Affiliations:** 1 Department of Clinical Epidemiology and Biostatistics, McMaster University, Hamilton, Canada; 2 Basel Institute for Clinical Epidemiology and Biostatistics, University Hospital Basel, Basel, Switzerland; 3 Knowledge and Encounter Research Unit, Mayo Clinic, Rochester, MN, USA; 4 Department of Neonatology, University Children's Hospital Tuebingen, Tuebingen, Germany; 5 Centre for Evidence-Based Medicine, Department of Primary Health Care, University of Oxford, Oxford, UK; 6 Universidad Peruana Cayetano Heredia, Lima, Peru; 7 State University of New York at Buffalo, Buffalo, NY, USA; 8 Cardiology Department, Vall d'Hebron Hospital, CIBER de Epidemiología y Salud Pública (CIBERESP), Spain; 9 Centro Cochrane Iberoamericano, Hospital Sant Pau, Barcelona, and CIBER de Epidemiologia y Salud Publica (CIBERESP), Spain; 10 Teaching and Research Center of Pro-Cardiaco, Rio de Janeiro, Brazil; 11 Pediatric Hematology and Oncology Centre for Pediatrics and Adolescent Medicine, University Hospital Freiburg, Freiburg, Germany; 12 Center for Evidence-based Medicine, USF Health Clinical Research, Tampa, FL, USA; 13 Sunnybrook Health Sciences Centre and University of Toronto, Toronto, ON, Canada; 14 British Columbia Centre for Excellence in HIV/AIDS, University of British Columbia, Vancouver, BC, Canada; 15 University of Western Ontario, London, ON, Canada; 16 Children's Hospital Philadelphia, Philadelphia, PA, USA; 17 Mount Sinai Medical Center, Miami Beach, FL, USA; 18 St. Michael's Hospital, Keenan Research Centre and Li Ka Shing Knowledge Institute, University of Toronto, Toronto, ON, Canada; 19 Department of Medicine, Gjøvik, Innlandet Hospital Health Authority, Norway; 20 Hospital Nacional de Geriatría y Gerontología San José, Costa Rica; 21 National School of Public Health (ENSP), Rio de Janeiro, Brazil; 22 Ottawa Hospital Research Institute, University of Ottawa, Ottawa, ON, Canada

## Abstract

**Background:**

Randomized clinical trials (RCTs) stopped early for benefit often receive great attention and affect clinical practice, but pose interpretational challenges for clinicians, researchers, and policy makers. Because the decision to stop the trial may arise from catching the treatment effect at a random high, truncated RCTs (tRCTs) may overestimate the true treatment effect. The **St**udy **O**f Trial **P**olicy Of **I**nterim **T**runcation (STOPIT-1), which systematically reviewed the epidemiology and reporting quality of tRCTs, found that such trials are becoming more common, but that reporting of stopping rules and decisions were often deficient. Most importantly, treatment effects were often implausibly large and inversely related to the number of the events accrued. The aim of STOPIT-2 is to determine the magnitude and determinants of possible bias introduced by stopping RCTs early for benefit.

**Methods/Design:**

We will use sensitive strategies to search for systematic reviews addressing the same clinical question as each of the tRCTs identified in STOPIT-1 and in a subsequent literature search. We will check all RCTs included in each systematic review to determine their similarity to the index tRCT in terms of participants, interventions, and outcome definition, and conduct new meta-analyses addressing the outcome that led to early termination of the tRCT. For each pair of tRCT and systematic review of corresponding non-tRCTs we will estimate the ratio of relative risks, and hence estimate the degree of bias. We will use hierarchical multivariable regression to determine the factors associated with the magnitude of this ratio. Factors explored will include the presence and quality of a stopping rule, the methodological quality of the trials, and the number of total events that had occurred at the time of truncation.

Finally, we will evaluate whether Bayesian methods using conservative informative priors to "regress to the mean" overoptimistic tRCTs can correct observed biases.

**Discussion:**

A better understanding of the extent to which tRCTs exaggerate treatment effects and of the factors associated with the magnitude of this bias can optimize trial design and data monitoring charters, and may aid in the interpretation of the results from trials stopped early for benefit.

## Background

Interim analyses conducted early in the course of a randomized clinical trial (RCTs) may suggest larger than expected treatment effects that are inconsistent with chance. Consequently, investigators and data monitoring committees may conclude that one treatment is superior to the other and decide to stop the trial and release the results, arguing that completing the trial as planned is unadvisable or even unethical. The publicity surrounding such action often captures considerable attention because of the large treatment effects and the dramatic nature of the decision to terminate early.

Clinicians, authors of systematic reviews and meta-analyses, and professional organizations issuing recommendations face challenges when interpreting the results of such truncated RCTs (tRCTs), because the results could be overly optimistic. Bias arises because random fluctuations towards greater treatment effects may result in early termination[1] In other words, the estimated treatment effect may be exaggerated because statistical stopping rules are prone to stop a trial at a random high. If the decision to stop the trial resulted from observing the apparent benefit of treatment at a random high, the resulting estimate of the treatment effect will be misleadingly large. If this occurs, data from future similar trials would be expected to yield a smaller estimate of treatment effect, as a consequence of the so-called "regression to the (true) mean" effect[2]

A systematic review of 143 RCTs stopped early for benefit, the **St**udy **O**f Trial **P**olicy Of **I**nterim **T**runcation (STOPIT)-1, was published in 2005[3] This systematic review documented a recent doubling in the number of tRCTs and found that tRCTs are often published in high profile medical journals. The reporting of the methods that informed the decision to truncate the trials was often deficient, and treatment effects were often implausibly large, especially when the number of events was small. A recent review focusing on oncology trials stopped early for benefit raised further concerns, finding that almost 80% of tRCTs published in the last three years were used to attain regulatory approval[4]

The extent to which tRCTs actually overestimate effects, the magnitude of bias, and the factors associated (and perhaps causally related) with the magnitude of bias in individual situations, remain uncertain. If one could identify a comprehensive set of trials not stopped early that addressed the same question as a tRCT, those trials or all trials (including the tRCT) could provide a least biased assessment of the true treatment effect that the corresponding tRCT (or tRCTs if there were more than one addressing the same question) was trying to estimate. The goal of this project is to obtain such a comprehensive set of trials matching a number of tRCTs, and thus address the question of magnitude of bias, and variables associated with the magnitude of bias.

### Study objectives

The present study, STOPIT-2, seeks to determine the magnitude and determinants of bias that truncation of RCTs for benefit may introduce. Our primary research questions are:

• *What is the extent to which tRCTs overestimate the treatment effect compared with the best available estimate of treatment effect as determined by a systematic review and meta-analysis of RCTs addressing the same question as the tRCT?*

• *What factors are associated with the size of the observed difference in the treatment effect between the tRCT and the respective meta-analytic estimate?*

• *Can Bayesian methods, using conservative priors, provide more likely estimates of the true underlying treatment effect, i.e. somehow "correct" for truncation bias?*

## Methods

### Overview of methods

STOPIT-1 included 143 tRCTs and we were able to identify 14 additional RCTs stopped early for benefit through a hand search in the medical literature and personal contact with trial investigators. The effort to identify tRCTs will continue by updating the search that led to the trials identified for STOPIT-1 (November 2004) using the same search strategy and through citation searching linked to the STOPIT-1 publication and accompanying editorial in JAMA[3,5]

**Figure 1:**
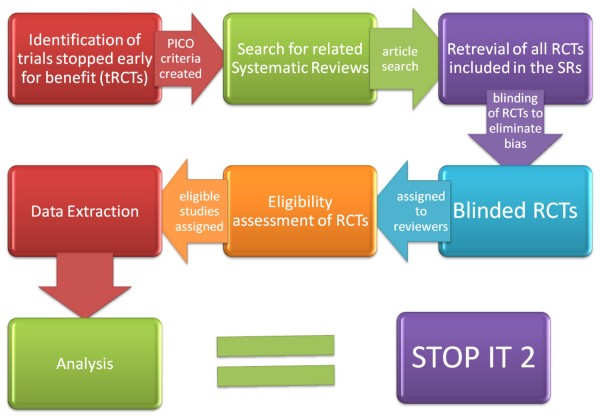
**Flow chart of the Study of Trial Policy of Interim Truncation (STOPIT)-2**. Abbreviations: RCT, randomized clinical trial; tRCT, truncated randomized trial due to stopping early for benefit; PICO, patient population, intervention, control, outcome.

In STOPIT-2, we will search for systematic reviews addressing the same question as the tRCTs (Figure 1). We will utilize the sensitive strategy for systematic reviews put forth for MEDLINE by Montori et al[6] Systematic reviews that ask a similar question to the tRCT but do not include the tRCT due to its publication after the search date of the systematic reviews will be updated to the present time. Other systematic reviews that include the tRCT will not be updated. Systematic reviews that are only similar under the broadest of definitions will be included only if the review authors chose to pool the tRCT within the systematic review.

From each eligible systematic review we will blind each RCT's results and two independent reviewers will determine eligibility. From each eligible trial we will then extract data and conduct new meta-analyses addressing the outcome that led to the early termination of the tRCT(s). First, we will compare the relative risk generated by the tRCT with the relative risk from all non-truncated studies. Second we will use multivariable regression to determine the factors associated with the difference in magnitude of effect between the tRCTs and RCTs not stopped early. These factors will include the presence and quality of a stopping rule, the methodological quality of the trials, and the number of events that had occurred at the time of truncation. Finally, we will compare possible methods for correcting the treatment effect estimates from tRCTs, in particular the use of Bayesian methods using conservative informative priors to "regress to the mean" the tRCT estimates. We will then compare the degree of disagreement with the meta-analytical estimates between the Bayesian-adjusted tRCT and the unadjusted tRCT results.

Some authors have suggested that pooling tRCTs with non-truncated trials addressing the same question will yield minimally biased estimates of treatment effects[7,8] However, our previous empirical finding that stopped-early studies contributed more than 40% of the weight in more than a third of meta-analyses including tRCTs challenges this view[9]) Nevertheless, if the overall estimate of treatment effect (based on all studies, including tRCTs) were the least biased estimate of the true underlying effect, it is this estimate to which one should compare tRCTs. Based on simulations and theoretical considerations we found compelling strengths and compelling limitations for each approach (Table 1). We will explore the extent to which results are consistent with the hypothesis that the pooled estimate including the tRCTs is least biased using our empirical data (for instance, the tRCTs should provide a relatively small weight in the meta-analysis as a result of their having fewer events because of stopping early). Choosing a primary analysis for a study commonly involves some arbitrariness. Given compelling reasons for either approach, we decided to conduct both analyses. We chose non-truncated RCTs only as the comparator in our primary analysis. In a complementary second analysis, we will compare the tRCT and the pooled estimate of all trials including the tRCT.

**Table 1:** Comparison of non-truncated RCTs only and truncated + non-truncated RCTs as comparators to estimate the magnitude of bias associated with stopping clinical trials early for benefit based on simulations and theoretical considerations.

**Non-truncated RCTs only**	**Truncated & non-truncated RCTs**
- more appropriate when the number of non-truncated RCTs in meta-analyses is relatively small (= weight of tRCTs in meta-analyses relatively large)	- more appropriate when the number of non-truncated RCTs in meta-analyses is relatively large (= weight of tRCTs in meta-analyses relatively small)

- more appropriate when true treatment effects are small (RCTs in meta-analyses likely to be underpowered)	- more appropriate when true treatment effects are large (RCTs in meta-analyses likely to be adequately powered)

- more appropriate in the presence of considerable publication bias	- more conservative bias estimation

- more appropriate when proportion of trials in meta-analyses without formal stopping rule is large	

- trial sample separate/independent from tRCT(s) facilitates statistical analysis	

Abbreviations: RCTs, randomized clinical trials; tRCT(s), truncated randomized clinical trial(s) due to stopping early for benefit

### Literature Search for Systematic Reviews

For meta-analyses, we will search the Cochrane Database of Systematic Reviews and the Database of Abstracts of Reviews of Effects using the population and intervention of the tRCTs as search terms. We will also search for meta-analyses in MEDLINE with textwords and Medical Subject Heading terms based on the study population and the intervention specified in the research question of the tRCTs, if necessary supplemented by a specified outcome, and with textwords "meta-analysis" OR "overview" OR "systematic review" and in a second approach with limits "meta-analysis.pt." AND "human"[6]

### Eligibility of Systematic Reviews

Systematic reviews will be considered eligible if they meet all of the following 5 criteria:

1) Report the methods used to conduct the review

2) Describe a literature search that, at minimum, includes MEDLINE

3) Include a population similar to that of the tRCT

4) Include an intervention similar to that of the tRCT

5) Include an outcome similar to the one that was the basis of the decision to stop the tRCT early

Because there is considerable judgment involved in the eligibility decisions, particularly criteria 3 to 5, every decision of the initial adjudicators will be reviewed and confirmed or refuted by another adjudicator and if necessary, by a third party. If in doubt while applying the broadest similarity criteria, a key factor for eligibility will be that the systematic review pooled the tRCT. In general, if in doubt, we will judge the systematic review eligible, because there will be a second review of eligibility at the level of individual trials.

### Updating of Systematic Reviews

The only systematic reviews we will update are those that did not include the index tRCT(s) because they were completed prior to the publication of the tRCT. In these instances, we will update the search of the systematic reviews to the present using the same strategy used in the systematic review. We will not update all meta-analyses in the systematic reviews, only the ones for the outcomes that led to the early termination of the matching tRCT(s).

### Identification, retrieval and eligibility of RCTs included in the systematic reviews

For each systematic review we will retrieve all included RCTs in full text (including associated manuscripts describing methods) to determine their similarity to the index tRCT. We will obtain data from unpublished studies that were included in the systematic reviews by contacting the authors of the systematic review and/or the authors of the unpublished studies. Including trials addressing a question that was different to that addressed in the relevant tRCT would bias the assessment of magnitude of effect from the trials not stopped early. Thus, we will judge the eligibility of each trial in the systematic review on the basis of the following criteria:

1) Including a population similar to that of the tRCT

2) Including an intervention similar to that of the tRCT

3) Including a control similar to that of the tRCT

4) Including an outcome similar to the one that led to the early termination of the tRCT

5) Random allocation to intervention and control group

One could have criteria for similarity that are very strict, or very permissive. As it is uncertain what the right approach is, we will classify the population, intervention, control and outcome of each potentially eligible trial as either "more or less identical", "similar, but not identical" or "broadly similar". The eligibility form will allow differentiation between eligibility of the studies based on the narrow, the broad or the broadest criteria and the "closeness" of the RCTs to the index tRCT will be considered in the analyses. We will construct a number of teams of two reviewers to make the eligibility decisions.

Each team will include individuals with expertise relevant to the content of the studies they will review. Within each pair of reviewers, the rating of the individual RCTs will be done independently and in duplicate. Disagreements will be resolved by discussion and, if necessary, by a third party. Because we are at risk of bias in the decision about whether to include a RCT based on the results, the reviewers who judge eligibility will be blinded to the results of the trial. Blinding will be accomplished by a separate team, not involved in study selection, using black ink on "hard copies" before these are scanned into electronic format or using black boxes overlaid on the sections describing results on electronic versions in portable document format of the paper. Every section of potentially eligible RCTs that reports the magnitude of results (abstract, results and discussion) will be blinded before the decision on eligibility is made. Blinding will be tested in a random set of 20 papers sent to 20 reviewers to ensure its success.

**Figure 2:**
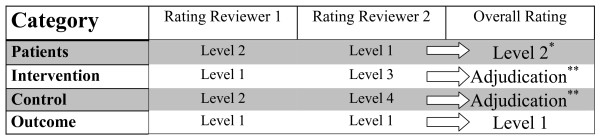
**Example to illustrate the process of judging similarity between a randomized clinical trial and the corresponding truncated randomized clinical trial**. Level 1 = Meets narrow criteria; Level 2 = Meets broad criteria; Level 3 = Meets broadest criteria; Level 4 = Does not meet criteria. * For differences in reviewer ratings of 1 level we will consider the broader similarity rating for the overall rating. ** Differences in reviewer ratings of 2 levels or greater will require adjudication by a third reviewer.

For RCTs, disagreements in relation to similarity of 2 levels or greater will require adjudication. Disagreements in relation to similarity of 1 level will not and the broader similarity rating will be assumed correct (Figure 2).

### Data extraction

From each RCT, we will collect the following data in duplicate.

1. Stopped early (yes/no)

2. Methodological quality: allocation concealment (documented as central independent randomization facility or numbered/coded medication containers prepared and distributed by an independent facility (e.g. pharmacy)); blinding of participants, care providers, and outcome adjudicators (blinding of participants and care providers will be rated as "probably yes" when trial report states "double blinded" or "placebo controlled"); loss to follow-up (we will collect the number of participants randomized and the number of participants with outcome data for the outcome of interest allowing for an estimation of loss to follow-up)

3. Measure of treatment effect for the outcome that terminated the tRCT (events and number randomized in intervention and control groups)

4. Pre-implemented stopping characteristics, if any (e.g., planned sample size, interim looks, stopping rules, number of events)

5. Date of conduct of the trial (start date, stop date, publication date)

### Statistical Analysis

We will calculate relative risks for each RCT in our study. For studies that provide results as continuous data (means, standard deviations), we will estimate an approximate dichotomous equivalent. To do this we will assume normal distributions of the results and that half a standard deviation represents the minimal important change[10] Using baseline data we will obtain the 0.5 standard deviation threshold from the baseline distribution and calculate the proportion of each follow-up distribution above or below (depending on the direction of the outcome) the threshold, i.e. the proportion of patients in each treatment arm who "did worse". This will allow us to specify relative risks and associated confidence intervals. If baseline data are not available, we will use the follow-up distribution of the control group to substitute for the 0.5 standard deviation threshold.

As well, for each meta-analysis we will calculate the pooled relative risk and 95% confidence interval for all trials that were not stopped early. Where there is more than one tRCT per meta-analysis we will also calculate a pooled relative risk and confidence interval for those tRCTs. These pooled estimates of relative risks will be calculated using an inverse variance weighted random effects model.

We will graphically present the results in a scatterplot of the effect size (relative risk) of the tRCT (horizontal axis) against the pooled effect size of non-tRCTs (vertical axis). If the tRCT and non-tRCTs give similar results, the points should be scattered along the diagonal of the scatterplot; if the tRCTs overestimate treatment effects they should be found above the diagonal.

We will also perform a z-test for each meta-analysis to look for differences between the truncated and non-truncated RCTs for the pooled relative risks. As a summary measure we will calculate a ratio of relative risks for each meta-analysis as follows:



We will plot the log(ratio of relative risks) and calculate an overall log(ratio of relative risks) as an inverse variance-weighted average of the log(ratio of relative risks). These will be back transformed and the ratio of relative risk values will be plotted for presentational purposes.

To investigate possible predictors of treatment effect sizes in RCTs, we will perform a hierarchical (multi-level) regression analysis. Our model will have two levels: individual RCT (study) level and meta-analysis level. The dependent variable in this analysis will be the logarithm of the relative risk (logRR) for each study and we will investigate the associations of the logRR with characteristics of the individual studies. We will investigate five possible predictors. Our main predictor of interest is a variable that we will construct from two different study characteristics, the presence and quality of a stopping rule and whether or not the RCT was truncated early.

The rule for stopping early will be categorized as one of three possibilities: (i) a rigorous rule (published prior to the trial plan), (ii) a not-so-rigorous rule such as *ad hoc *rules developed during the trial, (iii) no rule or unknown. Each of these three possibilities will be combined with whether or not the trial stopped early, creating 6 categories in total. It is very likely that there will be less than six categories in our final analysis as it is quite conceivable that some of the scenarios will not occur. We will carry out *post hoc *comparisons of outcomes between these 6 groups, focusing on contrasts that highlight the effects of the rule and the "truncated study" variable, and their interaction, to the extent that the available data permit.

Other study-level characteristics that we will examine are the methodological quality (blinding of patients, care-givers, and outcome assessors, and allocation concealment), and the total number of events. At the meta-analysis level, the only variable in the model will be an indicator of the specific meta-analyses to which each study belongs.

We will look at the main effects of all the variables and the interaction between the rule/truncated variable and the other predictor variables. Each study will yield a summary statistic (logRR) and an associated variance. The variance will provide weights for a meta-regression to evaluate the determinants of the estimated treatment effect.

The multivariable regression described above will be performed on 5 different datasets based on different levels of a variable which we will call *closeness*. This variable will measure how similar the non-truncated trials in each meta-analysis are to the corresponding truncated trial(s) with regard to the a) patient population, b) treatment arm, c) control arm and d) outcome. For each of these four, we will categorize closeness into one of three levels: very close (termed as 'fits the narrow criteria' in the database), moderately close (termed as 'fits the broad criteria' in the database), and less close (termed as 'fits the broadest criteria' in the database). This judgement will be coded by 2 reviewers, and the level of agreement (kappa) checked. Each trial will then be categorized by its least close category over the four areas which we will use to define our 5 different datasets. The datasets will be: 1) only trials that are "very close" in all domains; 2) trials with one or more "moderately close" domain, but no "less close" domains and not "very close" in all domains; 3) trials that are "less close" in at least one domain; 4) trials that are "very close" or "moderately close" in all domains (corresponds to 1) and 2) combined); and 5) all trials.

As discussed previously, we will conduct a further analysis in which the comparison is between the tRCT and the pooled estimate of all trials including the tRCT. If the tRCTs provide relatively small weights in the meta-analyses as a result of fewer events because of stopping early, the pooled estimate including the tRCTs may provide the least biased summary estimate.

Finally, we will compare possible methods for correcting the estimates from tRCTs for possible bias, in particular the use of Bayesian methods. The basic approach here is to use a conservative prior for trials (derived empirically from past trials in other areas – we will review such existing reviews [11-13]) and combine this information with the data from the tRCT to obtain a posterior estimate of effect. The weight will depend on the relative variance of the conservative prior and the tRCT: small trials will lead to an emphasis on the conservative prior whereas large trials will attach relatively greater importance to the observed data. We will calculate such Bayesian relative risks for each tRCT in our study. As for the simple tRCT estimate, we will graphically present the results for a visual comparison of the effect size (relative risk) of the truncated RCT(s) and the non-truncated RCTs. Based on previous simulation work [14] we would predict that the Bayesian estimates obtained will be closer to the meta-analysis findings.

## Discussion

The results of STOPIT-2 will extend earlier modeling studies and a systematic review of trials stopped early to provide a precise estimate of the extent of that bias as it plays out in the real world. Estimating the extent of bias and factors associated with the magnitude of bias will have implications for the design, conduct, and reporting of future clinical trials.

### Strengths and limitations of our protocol

Strengths of our study protocol include a systematic and extensive literature search with the goal of compiling a comprehensive sample of RCTs stopped early for benefit. Notwithstanding, despite complementing our electronic literature search of multiple databases with hand searches and personal contacts, we may fail to identify relevant tRCTs as truncation is often not explicitly stated in trial abstracts or methods[15]

We will use sensitive search strategies to identify systematic reviews corresponding to tRCTs,[6] and will undertake a labor-intensive process of judging eligibility of several thousand individual RCTs, each being assessed independently by two reviewers blinded to the RCT results. The blinding of trials prior to review is a particular strength of this study, limiting the potential for biased eligibility assessment on the basis of the magnitude of effect of the studies.

The accuracy of our results depends on the comprehensive search of the systematic reviews we will include. We have set a relatively low threshold for inclusion of corresponding systematic reviews: that the review included a methods section and that the systematic search included at least MEDLINE. Publication bias threatens all systematic reviews, and many do not include a thorough search for unpublished trials. To the extent that publication bias exists, however, it will likely lead to overestimates of the effects found in the pooled non-truncated RCTs. We assume that publication bias is less likely to affect the tRCTs. Thus, we anticipate that publication bias will yield an underestimate of the upward bias of the tRCTs relative to the non-tRCTs, and hence that our estimate of bias associated with truncation is likely to be conservative.

Despite objective criteria, when assessing the methodological quality of RCTs we are limited by the quality of the reporting of the trials.

A further strength of the study is the planned hierarchical analysis in which we link the estimates of treatment effect from tRCTs and non-tRCTs addressing the same question. Since the extent to which studies address the same question is a matter of judgment, the provision for a sensitivity analysis based on similarity of question will further strengthen the results.

### Ethical and data monitoring implications

The results of this study may have a profound effect on the decision-making of data monitoring committees[16] Data monitoring committees have an ethical obligation to ensure patients are offered effective treatment as soon as it is clear that an effective treatment is indeed available. To many people, this mandates a stopping rule that will ensure that any trial that shows an apparently large treatment effect at an early stage does not continue beyond a certain point. Data monitoring committees also have, however, an ethical obligation to future patients,[14] who require precise and accurate data addressing patient-important outcomes to make optimal treatment choices. While there is growing awareness that stopping trials early for apparent benefit may lead to overestimated results,[17] little is known about the magnitude and the determinants of bias that truncation introduces. The results of STOPIT-2 will provide valuable guidance to investigators, institutional review boards, funding agencies, and data monitoring committees regarding appropriate use of stopping rules in clinical trials.

### Public Engagement in Science

The results of STOPIT-2 will impact on numerous issues of public interest. Investigators, patients and their advocates, institutional review boards, and funding agencies may have different but convergent interests in stopping a study as soon as an important difference between experimental and control group emerges. Increased impact and publicity may motivate investigators, journals, and funding agents. A commitment to promptly offer participants in the less favorable arm the better treatment choice may motivate investigators, patients and their advocates, and data monitoring committees. The opportunity to save research dollars by truncating a trial may motivate the funding agencies.

Although a recent extension to the CONSORT statement for abstracts highlights the importance of reporting if the trial has stopped earlier than planned and the reason for doing so,[18] a number of observations suggest that investigators, journal editors, and clinical experts remain mostly unaware of the problematic inferences that may arise from truncated RCTs. Top journals continue to publish results of stopped early trials, but fail to require authors to note the early stopping in the abstract and to report details that would allow readers to carefully evaluate the decision to stop early[3] The QUOROM guidelines for meta-analyses and systematic reviews of RCTs [19] recommend that authors describe potential biases in the review process, but systematic reviewers pay scant attention to the potential bias introduced by including in their meta-analyses RCTs that were stopped early for benefit[9] Important professional organizations continue to issue recommendations on the basis of trials stopped early for benefit, including those that had reported very few endpoints and which therefore seem most likely to overestimate effects[20,21]

Empirical evidence of the magnitude and determinants of bias that truncation introduces will ensure that investigators, editors, authors of meta-analyses and guideline panels become appropriately cautious in their interpretation of stopped early trials. Ultimately, this will reduce the risk of prematurely translating unreliable study findings into clinical practice.

The findings of STOPIT-2 will influence recommendations on how to design and conduct RCTs and meta-analyses, and how to report their results, as summarized in the CONSORT [22] and QUOROM [19] guidelines. The results of the proposed study will also have implications for systematic reviews, including those who publish in the Cochrane Library. Grading the strength of recommendations and quality of evidence in clinical guidelines will also be influenced by the findings of STOPIT-2[23] Given the increasing frequency with which trials are stopped early, the results of STOPIT-2 will be of interest to numerous stakeholders including patients and their physicians, investigators, research ethics boards, funding agencies, journal editors, and policy makers.

## Abbreviations

RCTs: Randomized clinical trials; tRCTs: Truncated randomized clinical trials due to stopping early for benefit; STOPIT: Study of Trial Policy of Interim Truncation; MESH: Medical Subject Heading; logRR: Logarithm of the relative risk; CONSORT: Consolidated Standards of Reporting Trials; QUOROM: Quality of Reporting of Meta-analyses; PICO: Patient population, intervention, control, outcome.

## Competing interests

The authors declare that they have no competing interests.

## Authors' contributions

All listed authors contributed to the design of this protocol.
